# Inhibition of miR-21 improves pulmonary vascular responses in bronchopulmonary dysplasia by targeting the DDAH1/ADMA/NO pathway

**DOI:** 10.1515/med-2022-0584

**Published:** 2022-12-09

**Authors:** Ying Zhong, Zhiqun Zhang, Xiaoqing Chen

**Affiliations:** Department of Child Health Care, The First Affiliated Hospital of Nanjing Medical University, 368 Jiangdong North Road, Nanjing 210036, Jiangsu, China; Department of Neonatology, Affiliated Hangzhou First People’s Hospital, Zhejiang University School of Medicine, Hangzhou 310000, Zhejiang, China; Department of Pediatrics, The First Affiliated Hospital of Nanjing Medical University, Nanjing 210036, Jiangsu, China

**Keywords:** bronchopulmonary dysplasia, miR-21, DDAH1, ADMA, NO

## Abstract

miR-21 has been confirmed to be overexpressed in neonatal rat lungs with hyperoxia-mediated bronchopulmonary dysplasia (BPD). The specific function of miR-21 in BPD is still unclear. We established the hyperoxia-induced BPD rat model *in vivo* and the hyperoxia-induced pulmonary microvascular endothelial cells (PMVECs) model *in vitro*. Transwell assay was utilized to detect the migratory capability of PMVECs. Tube formation assay was utilized to measure angiogenesis ability. ELISA was utilized to test nitric oxide (NO) production and the intracellular and extracellular Asymmetric Dimethylarginine (ADMA) concentration. Furthermore, the interaction between miR-21 and dimethylarginine dimethylaminohydrolase 1 (DDAH1) was evaluated using luciferase reporter assay. We found that miR-21 expression in PMVECs was increased by hyperoxia stimulation. Inhibition of miR-21 improved the migratory and angiogenic activities of PMVECs and overexpression of miR-21 exerted the opposite effects. Furthermore, knockdown of miR-21 increased NO production and decreased intracellular and extracellular ADMA concentration in hyperoxia-treated PMVECs. Next we proved that miR-21 could bind to DDAH1 and negatively regulate its expression. Rescues assays showed that DDAH1 knockdown reversed the effects of miR-21 depletion on hyperoxia-mediated PMVEC functions, NO production, and ADMA concentration. Importantly, miR-21 downregulation restored alveolarization and vascular density in BPD rats. This study demonstrates that inhibition of miR-21 improves pulmonary vascular responses in BPD by targeting the DDAH1/ADMA/NO pathway.

## 1 Introduction

Bronchopulmonary dysplasia (BPD) is a leading cause of chronic respiratory morbidity among survivors of preterm birth with the greatest risk for those born at 23–30 weeks gestational age [1,2]. The epidemiology of BPD continues to demonstrate that birth weight and gestational age are the most predictive risk factors for developing BPD, and the frequency of BPD has been approximately 40% in surviving infants born at ≤28 weeks gestational age and about 30% in infants with birth weight <1,000 g during the 20 years from 1993 to 2012 [3]. While many drugs have been tried to prevent or attenuate BPD [4,5], no specific and effective treatment is available; therefore, this disease is still associated with high mortality and morbidity [6]. Despite improved neonatal care, the number of BPD cases due to this condition have not decreased [7], secondary to increased survival of infants of lower gestational ages. Damaged intrauterine lung development and post-partum injury can harm angiogenic capability and alveolar formation, leading to distal alveolar simplification [8]. In recent years, studies have found that disrupted vascularization is the main cause for alveolar simplification in BPD [9,10]. Importantly, it may lead to the development of pulmonary hypertension (PH) [11]. The pathophysiology of BPD-PH includes decreased pulmonary blood vessels, endothelial cell dysfunction, and increased remodeling of resistance pulmonary arteries [10]. Therefore, the in-depth study of the pathogenesis of BPD will help to develop more effective therapeutic targets and improve the survival of BPD patients.

MicroRNAs (miRNAs) are small, endogenous, non-coding RNAs of ∼22–26 nucleotides in length that function primarily as post-transcriptional regulators [12]. A flow of studies have demonstrated that miRNAs can modulate lots of pathophysiological processes, including cell proliferation, metabolism, and organ development [13,14]. They have been considered as promising candidates for novel targeted therapeutic approaches to lung diseases [14]. For example, miR-27a relieves acute lung injury in mice by modulation of TLR4/MyD88/NF-κB pathway [15]. Adrenomedullin regulated by miRNA-574-3p protects premature infants with BPD [16]. MiR-21 was one of the first miRNAs to be identified as transcribed by RNA polymerase II [17]. It has been found to be upregulated in many pathological conditions including cancer and cardiovascular diseases [18]. A previous study reported that miR-21 was upregulated in neonatal rat lungs in response to hyperoxia exposure [19]. MiR-21 was detected as a common miRNA that changed in chronic lung disease patients and in the hyperoxia exposed mice [20]. Other studies have documented an increase in miR-21 in hypoxia-induced pulmonary hypertensive mice and localization of miR-21 to distal small arteries in the animal and human hypertensive lungs [21,22]. A hypoxia-induced increase in miR-21 levels contributes to endothelial dysfunction associated with a reduction in NO/cGMP signaling [23]. Additionally, elevated miR-21 expression induced by oscillatory shear stress or oxidized low-density lipoprotein resulted in endothelial cell activation [24]. Overexpression of miR-21 promotes endothelial dysfunction in HUVECs treated with TNF-α [25]. These reports suggest the close association between elevated miR-21 and pulmonary hypertension (PH) and endothelial dysfunction.

The main purpose of this study was the investigation of the biological function and mechanism of miR-21 in BPD. We hypothesized that inhibition of miR-21 could improve pulmonary vascular responses in BPD. We established the hyperoxia-induced BPD rat model *in vivo* and the hyperoxia-induced pulmonary microvascular endothelial cells (PMVECs) *in vitro* to estimate the influence of miR-21 on pulmonary vascular development.

## 2 Materials and methods

### 2.1 Cell culture and treatment

Human PMVECs obtained from BeNa Culture Collection Co., Ltd (Beijing, China) were incubated in DMEM (Gibco, NY, USA) added with 10% FBS (Gibco) and 100 U/mL Penicillin-Streptomycin solution (Gibco). PMVECs exposed to room air (RA) (21% O_2_/5% CO_2_/74% N_2_) served as the negative control (NC) group cells. PMVECs in the hyperoxia treatment group were exposed to high O_2_ gas (85% O_2_/5% CO_2_/10% N_2_). PMVECs were treated with hyperoxia (4, 16, and 24 h).

### 2.2 Cell transfection

Specific siRNAs against dimethylarginine dimethylaminohydrolase 1 (si-DDAH1) and its corresponding negative control (si-NC) (30 nM) were obtained from Genechem (Shanghai, China). miR-21 inhibitor, miR-21 mimic, NC inhibitor, and NC mimic (50 nM) were obtained from RiboBio (Guangzhou, China). PMVECs were cultured in RA or under hyperoxia for 24 h. Then, cell transfection was conducted for 48 h using Lipofectamine 3000 (Invitrogen, Carlsbad, CA) according to user guides. After 48 h, cells were collected, and the transfection efficiency was tested through RT-qPCR.

### 2.3 RT-qPCR

Total RNA was extracted from PMVECs utilizing TRIzol Reagent (Thermo Fisher Scientific, Waltham, MA, USA) and cDNA was synthesized from 1 μg of total RNA by PrimeScript^TM^ RT reagent Kit with gDNA (Takara, Japan). Then, qPCR was performed on StepOnePlus Real-Time PCR System (Applied Biosystems, USA) with SYBR Premix ExTaq^TM^ II (Takara). The expression of miR-21 or DDAH1 was calculated by the 2^−ΔΔCt^ method normalized to U6 or GAPDH, respectively. The sequences of primers used are as follows:

miR-21 forward, 5′-GCGGCAACACCAGTCGATG-3′,

miR-21 reverse, 5′-GCGGCAACACCAGTCGATG-3′;

DDAH1 forward, 5′-ACGTCCTATTCACAGGCAG-3′,

DDAH1 reverse, 5′-TATCAGCCAAGATCTCAGCA-3′;

GAPDH forward, 5′-AACTCCCATTCTTCCACCT-3′,

GAPDH reverse, 5′-TTGTCATACCAGGAAATGAGC-3′;

U6 forward, 5′-CAGTTATGACGACCTAGACAG-3′,

U6 reverse, 5′-CAAATTTGCATGTCATCCTTGG-3′.

### 2.4 Transwell assay

PMVEC migration was estimated using 8.0 µm Transwell inserts (Corning Inc., Corning, NY, USA). The 1 × 10^5^ PMVECs in 200 µL of serum-free medium were seeded on the upper chamber. Then, 900 µL of DMEM were filled in the lower chamber. After 24 h, cells in the top surface of the membrane were wiped off, and cells in the lower surface were fixed with 4% paraformaldehyde and dyed with crystal violet (Solarb, China) for 15 min. Next cells were washed with PBS three times, and examined by the microscope (Olympus, Japan).

### 2.5 Tube formation assay

For determining angiogenesis capacity *in vitro*, PMVECs (1 × 10^4^ cells/well) were seeded in 96-well plates coated by Matrigel (Corning Inc.) and cultured in different conditions. The culture medium was changed every 24 h and cells were cultured for 72 h. The capillary-network formation was detected using an inverted microscope (Logos Biosystem, Villeneuve d’Ascq, France). The quantity of the nodes in three randomly chosen fields of each plate was examined.

### 2.6 ELISA

Cells were rinsed with pre-cold PBS and cultured with 200 μL of cell lysis buffer at 4°C for half an hour. Then, samples were subjected to centrifugation at 12,000 rpm for 10 min and the supernatant was collected and maintained at −80°C. The BCA kit was applied for determining cell lysate concentration. The concentration of ADMA in cell medium and lysates was evaluated using ELISA kits (Cusabio, Wuhan, China) in accordance with user guides. DDAH metabolic activity and the concentration of NO were measured using their corresponding ELISA kits (Cusabio) following the manufacturer’s instructions.

### 2.7 Bioinformatics analysis

The target genes of miR-21 were predicted from the websites including starBase (https://starbase.sysu.edu.cn/index.php), miRDB (http://www.mirdb.org/mirdb/index.html), and miRWalk (http://mirwalk.umm.uni-heidelberg.de/).

### 2.8 Luciferase reporter assay

The putative binding site of miR-21 in the DDAH1 3′UTR was predicted by starBase database. DDAH1 3′UTR fragments containing the binding site with miR-21 were inserted in the pmirGLO luciferase reporter vector (Promega, Madison, Wisconsin, USA). Cells treated with RA or 24 h of hyperoxia were subjected to co-transfection with pmirGLO OTX1 3′UTR luciferase reporter vector and miR-21 inhibitor or NC inhibitor with Lipofectamine 3000 (Invitrogen) for 48 h. Luciferase activity was measured by Dual-Luciferase Reporter Assay System (Promega). The luciferase activity of Firefly was normalized to that of Renilla.

### 2.9 Establishment of hyperoxia-induced BPD newborn rat models

All procedures and protocols of this study were approved by the Institutional Animal Care and Use Committee of Zhejiang University (Zhejiang, China) A total of 8 SPF pregnant Sprague–Dawley rats (gestational age of 15 days; Vital River Co. Ltd, Beijing, China) were housed in a light- and temperature-controlled room with free access to food and water. Pregnant rats were raised alone and they gave birth by themselves after 1 week. After delivery, 48 newborn rats born 12 h apart were randomly divided into hyperoxia treatment group (*n* = 36) and control group (*n* = 12). Rats in the hyperoxia group were divided into the BPD group (BPD rats) (*n* = 12), the BPD + antagomir NC group (*n* = 12), and the BPD + miR-21 antagomir group (*n* = 12). The 85% oxygen concentration was utilized for hyperoxia group. Rats in the control group were maintained in RA (21% oxygen). The newborn rats were raised for 1 week, and then injected with the antagomir NC and miR-21 antagomir (20 mg/kg/day; 0.2 mL/day; GenePharma, Shanghai, China) by the tail vein. The control rats (air group) were fed in a normal pressure room (21% oxygen), and the newborn rats used for BPD model in the hyperoxia group were raised in a plexiglass oxygen chamber (85% oxygen). The cage was opened for half an hour every day and water and food were provided. The maternal rats were utilized for feeding the newborn rats and were exchanged among cages every 24 h. On the 1st, 3rd, 7th, and 14th days, rats were separately anesthetized with intraperitoneal injection of 90 mg/kg pentobarbital sodium, and then they were euthanized by cervical dislocation. Next the abdominal cavity was opened quickly to remove the lungs. After rapid freezing with liquid nitrogen, the lungs were stored in a −80°C refrigerator for subsequent assays.

### 2.10 Histological analysis

After the rats were executed on the 14th day, lung tissues were collected and cut into 5-μm-thick sections, followed by fixing with 4% paraformaldehyde for 48 h. The slices were dehydrated with graded ethyl alcohol solutions. Following xylene dewaxing and hydration, the slices were dyed with hematoxylin (Solarbio, Beijing, China) and eosin for 5 min. The morphological changes were analyzed using a light microscope (Eclipse Ci; Nikon, Tokyo, Japan). In accordance with the previously described method [26], it was also necessary to detect the radial alveolar count and alveolar area/pulmonary septal area value.

### 2.11 Immunofluorescence staining

Pulmonary vessel density was quantified based on immunofluorescence staining of the endothelial-specific marker von Willebrand factor (vWF) in rat lung section. Briefly, 5 μm sized paraffin-embedded lung sections were stained overnight with a primary antibody against vWF (1:250, AB7356, Sigma-Aldrich) at 4°C. Sections were stained with an Alexa Fluor 594-conjugated secondary antibody (1:200; Jackson Immunoresearch) at room temperature for 2 h. After a final washing step, sections were mounted with DAPI (Sigma-Aldrich) and quantified for vessel density by a blinded observer. Using ImageJ, the vWF-positive vessels (<100 μm diameter) were counted in five 10× magnified fields per subject and averaged.

### 2.12 Western blot

The lung tissues or cells were collected and homogenized in RIPA lysis buffer (Roche Applied Science, Indianapolis, IN). The protein concentration was tested using a BCA protein assay kit (Beyotime, China). Then, 15 μg/lane samples were loaded onto a 4–20% gel, resolved by SDS-PAGE and then transferred to PVDF membranes (Millipore, USA). The membranes were blockaded with 5% skimmed milk for 2 h to block non-specific binding. The PVDF membranes were mixed with primary antibodies and incubated at 4°C overnight. The next day, after rinsing with TBST, the membranes were cultured with the HRP-conjugated secondary antibody (Abcam, ab6789, 1:2,000) for 2 h at room temperature. The band density was tested using Quantity One software with an ECL kit (BioRad Laboratories, Shanghai, China). The primary antibodies used were: p-eNOS (ser1177) (PA5-35879, 1:500, Thermo Fisher Scientific, USA), DDAH1 (sc-271337, 1:1,000, Santa Cruz Biotechnology, Inc., USA), and β-actin (ab8226, 1:1,000, Abcam) served as loading control.

### 2.13 Statistical analysis

Statistical analyses were performed using GraphPad Prism 8 software (GraphPad Software, Inc., La Jolla, CA, USA). Group difference was analyzed by Student’s *t*-test or one-way ANOVA followed by Tukey’s *post hoc* analysis. Data are presented as mean value ± SD from three individual repeats. *p* < 0.05 was considered statistically significant.

## 3 Results

### 3.1 Inhibition of miR-21 improves the angiogenic activity of PMVECs

**Figure 1:**
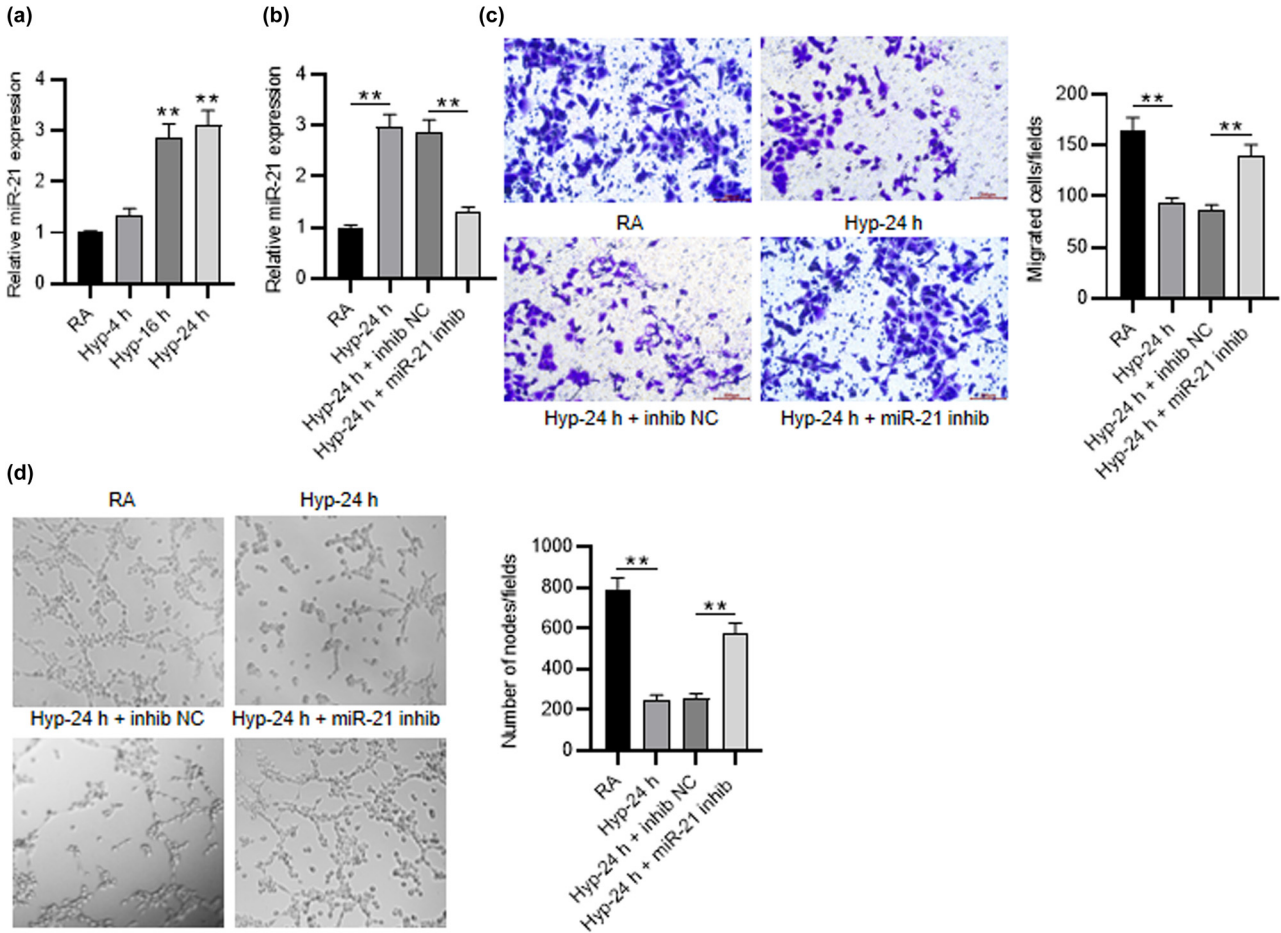
Inhibition of miR-21 improves the angiogenic activity of PMVECs. (a) RT-qPCR was used to measure miR-21 expression in PMVECs treated with RA or hyperoxia (4, 16 and 24 h). (b) The knockdown efficiency of miR-21 inhibitor in PMVECs treated with 24 h of hyperoxia was detected by RT-qPCR. (c) Transwell assay was applied for measuring the migration of hyperoxia-induced PMVECs when miR-21 was silenced. (d) Tube formation assay was performed to estimate the angiogenic activity of hyperoxia-induced PMVECs after miR-21 inhibition. Quantified values are mean values ± standard deviation of at least three independent experiments. ^**^ *p* < 0.01.

First, miR-21 expression in PMVECs with the treatment of RA or hyperoxia (4, 16, and 24 h) was tested by RT-qPCR. We found that, in comparison of RA treatment, miR-21 expression in PMVECs was notably increased after hyperoxia treatment in a time-dependent manner (*p* < 0.01) (Figure 1a). Then, we silenced miR-21 expression in PMVECs treated with hyperoxia-24 h by transfecting miR-21 inhibitor, and then found that miR-21 expression was downregulated after transfection (2.88 ± 0.23 vs 1.29 ± 0.11, *p* < 0.01) (Figure 1b). Transwell assay was conducted to measure the influence of miR-21 inhibition on the migration of PMVECs under hyperoxia. We discovered that hyperoxia inducement significantly repressed cell migration (285.00 ± 22.00 vs 89.00 ± 8.20, *p* < 0.01), while miR-21 depletion restored the migration of PMVECs (78.00 ± 7.00 vs 199.00 ± 12.00, *p* < 0.01) (Figure 1c). Next the tube formation assay was utilized to determine the angiogenic activity of PMVECs. The results demonstrated that miR-21 depletion restored the decreased number of nodes caused by hyperoxia inducement in PMVECs (255.00 ± 23.46 vs 573.00 ± 51.18, *p* < 0.01) (Figure 1d). These data suggested that miR-21 knockdown improves the migratory and angiogenic activity of PMVECs under hyperoxia treatment.

### 3.2 Knockdown of miR-21 increases NO production and decreases intracellular and extracellular ADMA concentration

**Figure 2:**
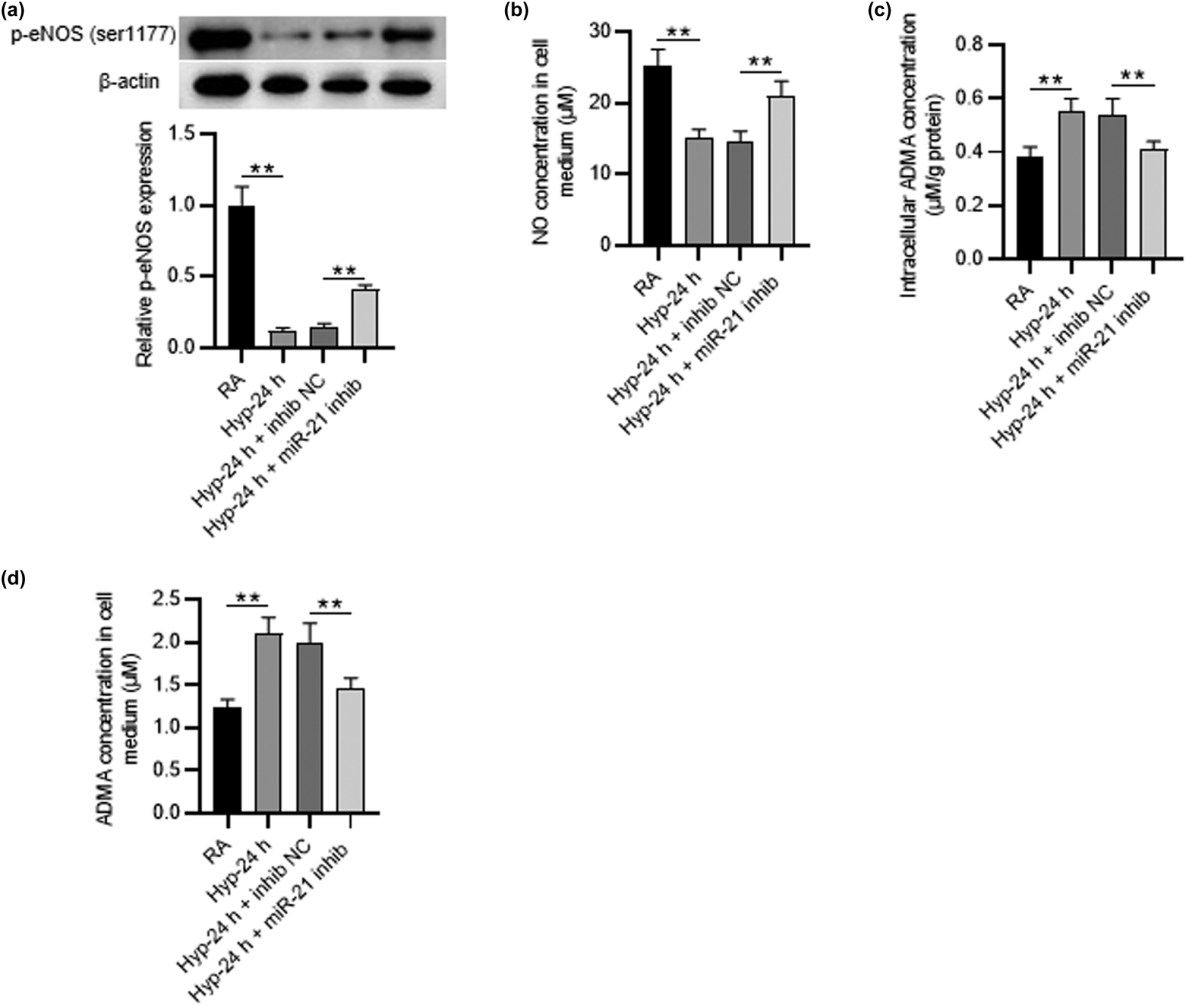
Knockdown of miR-21 increases NO production and decreases intracellular and extracellular ADMA concentration. (a) Western blot was utilized to test the p-eNOS (ser1177) level in PMVECs of different groups (RA group, hyperoxia-24 h group, hyperoxia-24 h + NC inhibitor group, hyperoxia-24 h + miR-21 inhibitor group). (b–d) ELISA was utilized to detect the NO production and the intracellular and extracellular ADMA concentration in PMVECs of different groups. Quantified values are mean values ± standard deviation of at least three independent experiments. ^**^ *p* < 0.01.

Nitric oxide (NO) is an endogenous pulmonary vasodilator produced by endothelial NO synthase [27,28]. NO plays an important role in maintaining endothelial function, and its production is mainly mediated by Asymmetric Dimethylarginine (ADMA) [29,30]. Thus, the concentrations of NO and ADMA in PMVECs under hyperoxia were detected. According to western blot, we observed that hyperoxia treatment markedly inhibited eNOS (ser1177) phosphorylation in PMVECs (1.00 ± 0.13 vs 0.12 ± 0.02, *p* < 0.01), while it was increased by miR-21 knockdown (0.15 ± 0.02 vs 0.41 ± 0.03, *p* < 0.01) (Figure 2a). ELISA further presented that NO concentration was reduced by hyperoxia treatment and was elevated by miR-21 depletion (14.67 ± 1.35 vs 20.96 ± 2.11, *p* < 0.01) (Figure 2b). Additionally, we found that hyperoxia treatment elevated the intracellular and extracellular ADMA concentrations in PMVECs (0.38 ± 0.04 vs 0.55 ± 0.05, 1.23 ± 0.10 vs 2.10 ± 0.19, respectively, *p* < 0.01). However, miR-21 depletion decreased both intracellular and extracellular ADMA concentrations in hyperoxia-induced PMVECs (0.54 ± 0.06 vs 0.41 ± 0.03, 2.00 ± 0.22 vs 1.46 ± 0.12, respectively, *p* < 0.01) (Figure 2c and d). Overall, miR-21 knockdown improves hyperoxia-induced PMVECs dysfunction via repressing ADMA concentration and elevating NO production.

### 3.3 Overexpression of miR-21 strengthens the effects of hyperoxia in PMVECs

**Figure 3:**
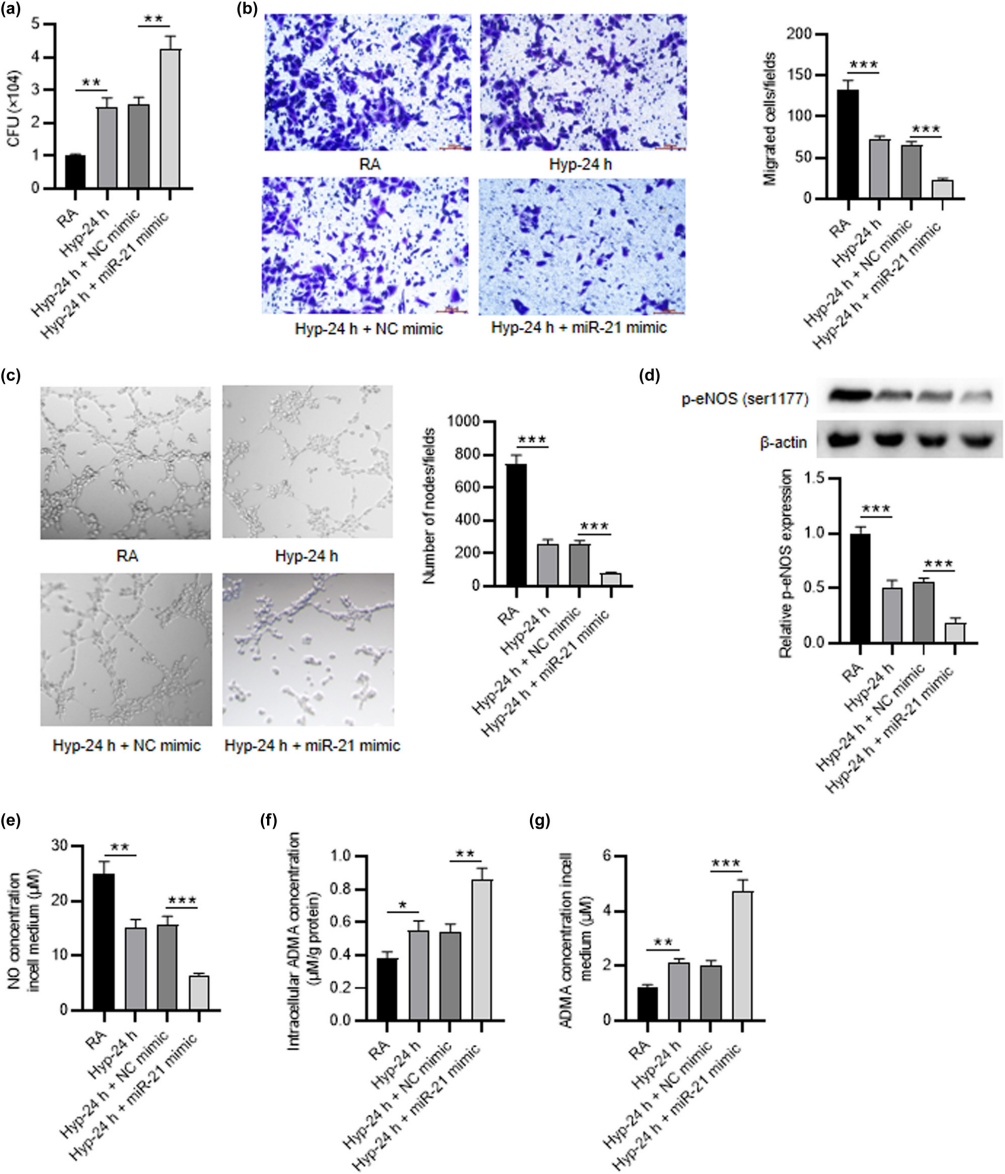
Overexpression of miR-21 strengthens the effects of hyperoxia in PMVECs. (a) The overexpression efficiency of miR-21 mimic in PMVECs treated with 24 h of hyperoxia was detected by RT-qPCR. (b) Transwell assay was applied to detect the migration of hyperoxia-induced PMVECs after miR-21 overexpression. (c) Tube formation assay was performed to estimate the angiogenic activity of hyperoxia-induced PMVECs after miR-21 overexpression. (d) Western blot was utilized to test the p-eNOS (ser1177) level in PMVECs of different groups (RA group, hyperoxia-24 h group, hyperoxia-24 h + NC mimic group, hyperoxia-24 h + miR-21 mimic group). (e–g) ELISA was utilized to detect the NO production and the intracellular and extracellular ADMA concentration in PMVECs of different groups. Quantified values are mean values ± standard deviation of at least three independent experiments. ^**^ *p* < 0.01, ^***^ *p* < 0.001.

To obtain more reliable results, we used miR-21 mimic to detect the impact of miR-21 overexpression on PMVECs. PCR results showed that miR-21 expression was upregulated in hyperoxia-treated PMVECs after miR-21 mimic transfection (2.55 ± 0.23 vs 4.26 ± 0.38, *p* < 0.01) (Figure 3a). Transwell and tube formation assays demonstrated that miR-21 overexpression strengthened the inhibitory effects of hyperoxia on cell migration (45.27 ± 4.23 vs 16.19 ± 1.46, *p* < 0.001) and angiogenesis (255.96 ± 21.33 vs 76.80 ± 7.42, *p* < 0.001) (Figure 3b and c). Western blot revealed that miR-21 mimic inhibited eNOS (ser1177) phosphorylation in hyperoxia-treated PMVECs (0.55 ± 0.04 vs 0.19 ± 0.04, *p* < 0.001), (Figure 3d). NO concentration was further reduced by miR-21 depletion (15.63 ± 1.54 vs 6.26 ± 0.55, *p* < 0.001) (Figure 3e). Furthermore, miR-21 depletion decreased both intracellular and extracellular ADMA concentrations in hyperoxia-induced PMVECs (0.54 ± 0.05 vs 0.86 ± 0.07, 2.00 ± 0.19 vs 4.70 ± 0.45, respectively, *p* < 0.01) (Figure 3f and g). These results demonstrated that miR-21 overexpression promotes hyperoxia-induced PMVECs dysfunction.

### 3.4 miR-21 negatively regulates DDAH1

**Figure 4:**
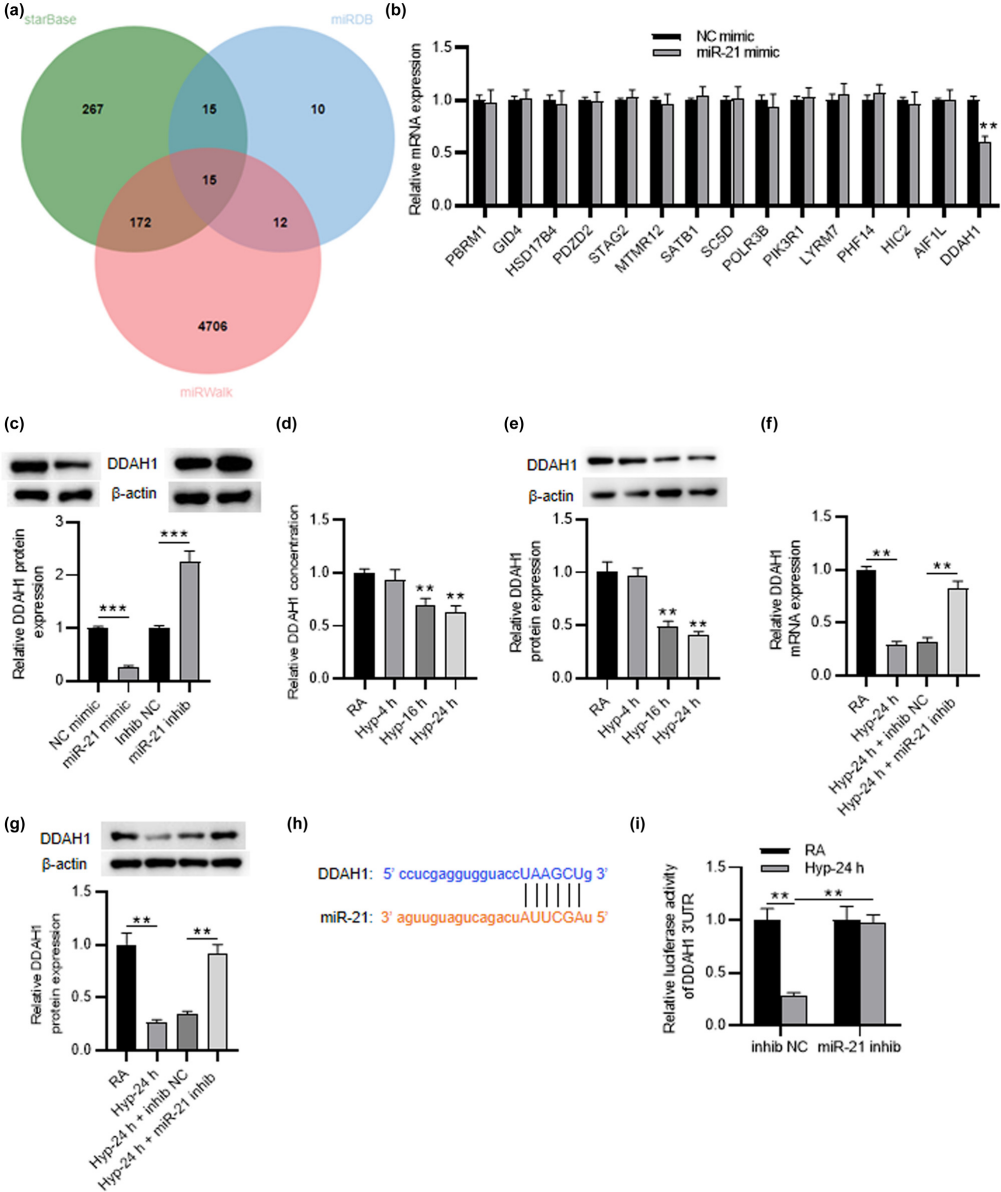
MiR-21 negatively regulates DDAH1. (a) Venn diagram showing the prediction results of target genes from starBase, miRDB, and miRWalk databases. (b) The mRNA expression of candidates in miR-21 mimic-transfected PMVECs was measured by RT-qPCR. (c) DDAH1 protein expression in PMVECs transfected with miR-21 mimic or inhibitor was measured by western blot. (d and e) RT-qPCR and western blot were used to measure DDAH1 expression in PMVECs treated with RA or hyperoxia (4, 16 and 24 h). (f and g) DDAH1 expression in PMVECs of different groups (RA group, hyperoxia-24 h group, hyperoxia-24 h + NC inhibitor group, hyperoxia-24 h + miR-21 inhibitor group). (h) The binding site of DDAH1 and miR-21. (i) Luciferase reporter assay was performed to verify the interaction of DDAH1 and miR-21 in PMVECs with RA or 24 h of hyperoxia. Quantified values are mean values ± standard deviation of at least three independent experiments. ^**^ *p* < 0.01, ^***^ *p* s< 0.001.

The targets of miR-21 were identified by utilizing bioinformatics prediction websites including starBase, miRDB, and miRWalk. Venn diagram shows 15 common target genes (PBRM1, GID4, HSD17B4, PDZD2, STAG2, MTMR12, SATB1, SC5D, POLR3B, PIK3R1, LYRM7, PHF14, HIC2, AIF1L, and DDAH1) predicted by these databases (Figure 4a). RT-qPCR showed that only DDAH1 was significantly downregulated in PMVECs transfected with miR-21 mimic (1.00 ± 0.04 vs 0.61 ± 0.05, *p* < 0.01) (Figure 4b). However, the other genes had no significant change. Studies have indicated that DDAH depletion is closely correlated with endothelial dysfunction and it is believed to be the mechanism responsible for ADMA-mediated eNOS impairments [31,32]. DDAH1 has been indicated to exert the vital function in regulating NO‐mediated apoptosis and angiogenesis in PMVECs [33]. Thus, we detected the interaction of DDAH1 and miR-21. The protein expression of DDAH1 was downregulated by miR-21 inhibitor and was upregulated by miR-21 mimic compared to control (1.00 ± 0.03 vs 0.26 ± 0.03, 1.00 ± 0.05 vs 2.25 ± 0.21, respectively, *p* < 0.001) (Figure 4c). RT-qPCR and western blot results illustrated that the mRNA and protein levels of DDAH1 were decreased in hyperoxia-induced PMVECs in a time-dependent manner (*p* < 0.01) (Figure 4d and e). Additionally, DDAH1 levels inhibited by hyperoxia treatment were increased by miR-21 knockdown (0.32 ± 0.04 vs 0.82 ± 0.07, 0.34 ± 0.03 vs 0.92 ± 0.08, respectively, *p* < 0.01) (Figure 4f and g). Furthermore, through starBase database, we obtained the binding site of miR-21 and DDAH1 (Figure 4h). The luciferase activity of DDAH1 3'UTR was almost unchanged in RA-treated PMVECs, but it reduced in hyperoxia-induced PMVECs (1.00 ± 0.11 vs 0.28 ± 0.03, *p* < 0.01). However, miR-21 knockdown elevated its luciferase activity in hyperoxia-induced PMVECs (0.28 ± 0.03 vs 0.97 ± 0.08, *p* < 0.01) (Figure 4i). Overall, miR-21 negatively regulates DDAH1 in hyperoxia-induced PMVECs.

### 3.5 DDAH1 knockdown reverses the effects of miR-21 depletion on hyperoxia-induced PMVEC behaviors, NO production, and ADMA concentration

**Figure 5:**
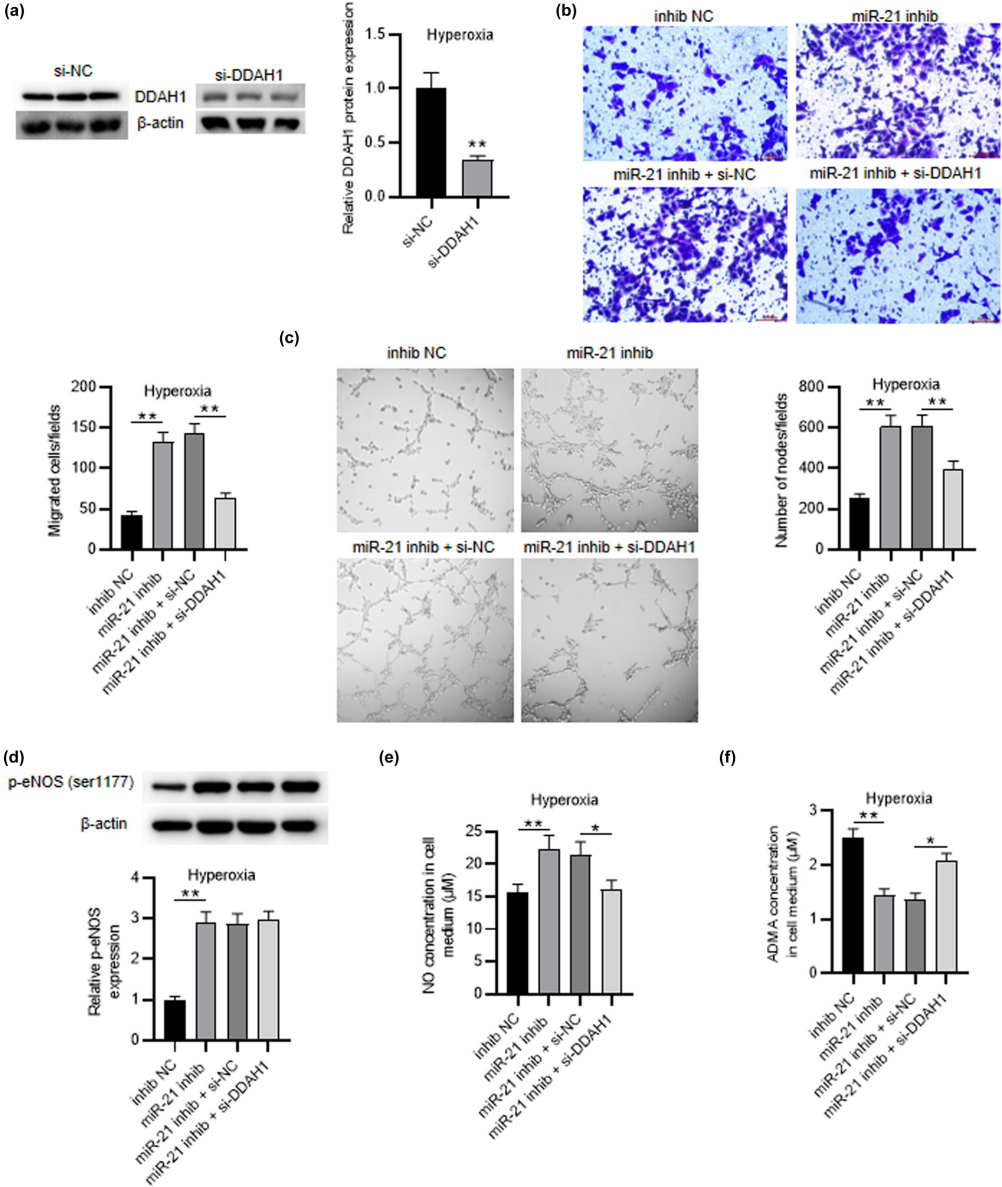
DDAH1 knockdown reverses the effects of miR-21 depletion on hyperoxia-induced PMVEC behaviors, NO production, and ADMA concentration. (a) Western blot was performed to test the knockdown efficiency of si-DDAH1 in hyperoxia-induced PMVECs. (b) Transwell assay was applied for measuring the migration of hyperoxia-induced PMVECs in different groups (NC inhibitor group, miR-21 inhibitor group, miR-21 inhibitor + si-NC group, miR-21 inhibitor + si-DDAH1 group). (c) Tube formation assay was performed to estimate the angiogenic activity of hyperoxia-induced PMVECs in different groups. (d) Western blot was utilized to test the p-eNOS (ser1177) level in hyperoxia-induced PMVECs of different groups. (e and f) ELISA was utilized to detect the NO production and the extracellular ADMA concentration in hyperoxia-induced PMVECs of different groups. Quantified values are mean values ± standard deviation of at least three independent experiments. ^*^ *p* < 0.05, ^**^ *p* < 0.01.

For the sake of validating the interaction between DDAH1 and miR-21 in hyperoxia-induced PMVECs, we performed a series of rescue assays. DDAH1 protein expression was inhibited in hyperoxia-induced PMVECs by transfecting with si-DDAH1 (1.00 ± 0.15 vs 0.34 ± 0.04, *p* < 0.01) (Figure 5a). Then, it was illustrated from transwell assays that cell migration promoted by miR-21 depletion was reduced by si-DDAH1 transfection (221.67 ± 20.25 vs 102.43 ± 8.76, *p* < 0.01) (Figure 5b). Furthermore, tube formation assay indicated that cell angiogenic activity enhanced by miR-21 depletion was weakened by DDAH1 silencing (612.33 ± 50.29 vs 398.64 ± 37.16, *p* < 0.01) (Figure 5c). Next we discovered that miR-21 knockdown markedly elevated p-eNOS level in cells, but DDAH1 silencing did not affect its level (2.87 ± 0.25 vs 2.96 ± 0.22) (Figure 5d). Moreover, ELISA results illustrated that NO concentration elevated by miR-21 depletion was reduced by DDAH1 downregulation (21.41 ± 2.03 vs 16.17 ± 1.31, *p* < 0.05) (Figure 5e). Additionally, the extracellular ADMA concentration decreased by miR-21 depletion was recovered by DDAH1 inhibition (1.36 ± 0.12 vs 2.08 ± 0.14, *p* < 0.05) (Figure 5f). Overall, DDAH1 knockdown reverses the effects of miR-21 depletion on hyperoxia-induced PMVEC behaviors, NO production, and ADMA concentration.

### 3.6 miR-21 is upregulated and DDAH1 is downregulated in the lung of BPD rats

**Figure 6:**
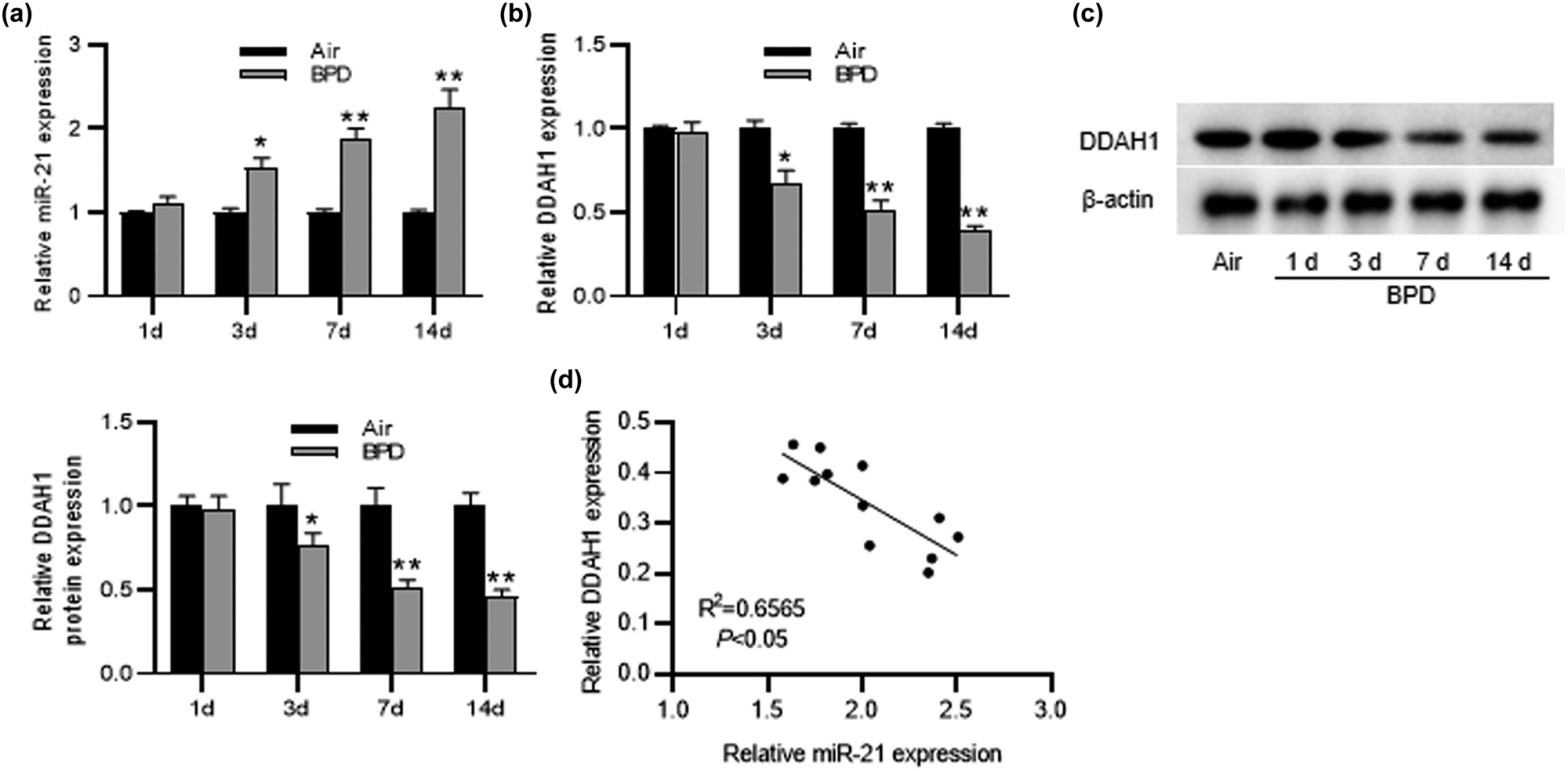
MiR-21 is upregulated and DDAH1 is downregulated in lung tissues of BPD rats. (a) MiR-21 expression was tested by RT-qPCR in lung tissues of rats treated with air or hyperoxia. (b and c) DDAH1 mRNA and protein expression in lung tissues of rats of different groups. (d) Pearson’s correlation analysis showing the correlation of miR-21 expression and DDAH1 expression in lung tissues of BPD rats. *N* = 12. Quantified values are mean values ± standard deviation of at least three independent experiments. ^*^ *p* < 0.05, ^**^ *p* < 0.01.

We performed the animal assays to further verify the correlation of miR-21 and DDAH1 *in vivo*. We established the BPD rat model by treating newborn rats with hyperoxia and obtained the lung tissues of rats on the indicated day and detected miR-21 and DDAH1 expression in tissues. RT-qPCR results indicated that, in comparison to air group, miR-21 expression was upregulated and DDAH1 expression was downregulated in lung tissues of BPD rats (1.00 ± 0.03 vs 2.26 ± 0.21, 1.00 ± 0.03 vs 0.39 ± 0.03, respectively, *p* < 0.01) (Figure 6a and b). Then, western blot further illustrated the downregulation of DDAH1 protein level in lung tissues of BPD rats (1.00 ± 0.08 vs 0.46 ± 0.94, *p* < 0.01) (Figure 6c). Furthermore, Pearson’s correlation analysis indicated that DDAH1 expression was negatively correlated with miR-21 expression in lung tissues of BPD rats (Figure 6d).

### 3.7 miR-21 antagomir restores alveolarization and vascular density in neonatal rats with BPD

**Figure 7:**
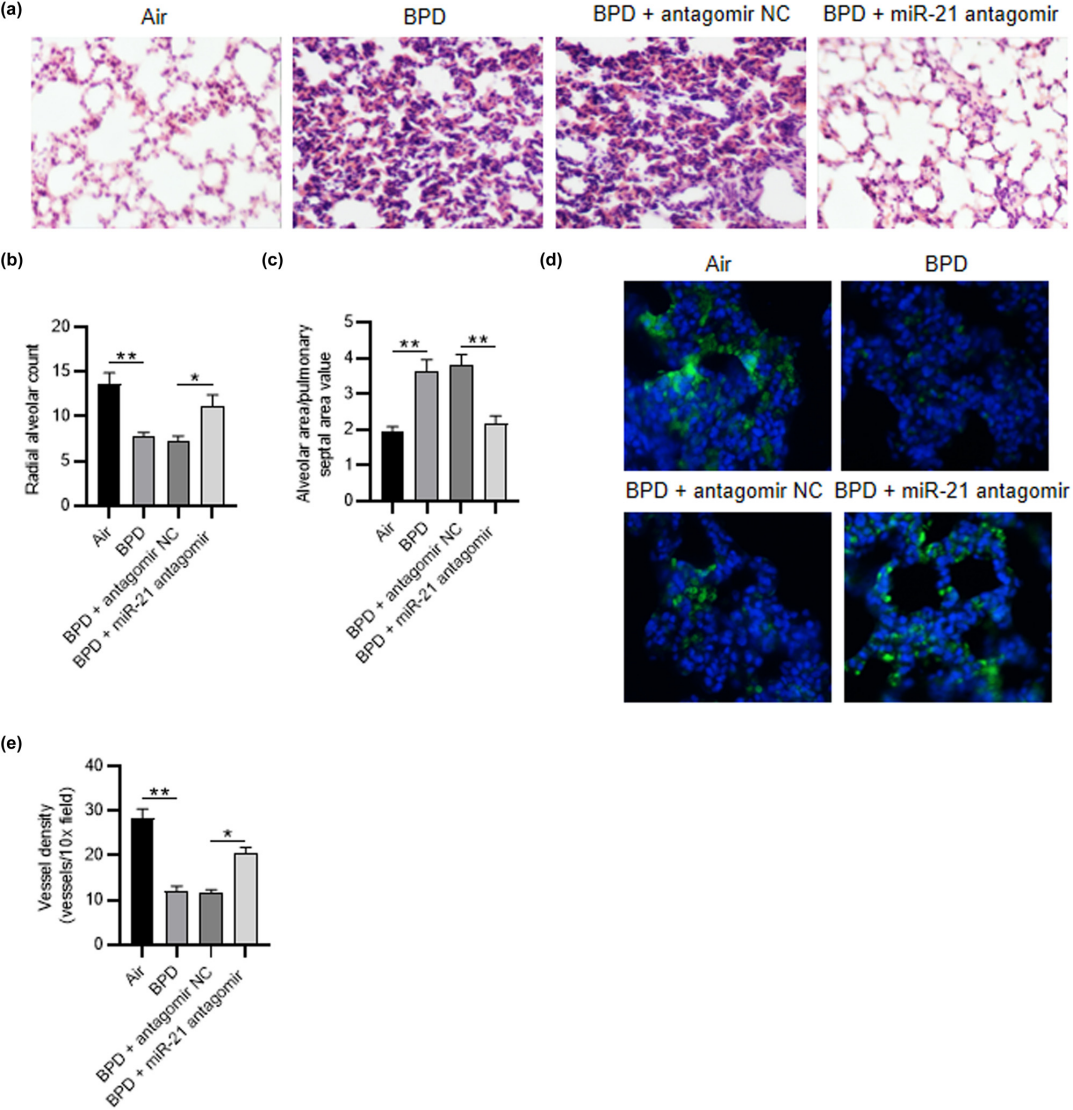
MiR-21 antagomir restores alveolarization and vascular density in neonatal rats with BPD. (a) H&E staining assay was utilized to detect the histopathological alterations of rats in different groups (air group, BPD group, BPD + NC antagomir group, BPD + miR-21 antagomir group). (b and c) The radial alveolar count and the alveolar area/pulmonary septal area value were detected. (d and e) Immunofluorescence staining was applied for measuring the vWF-positive vessels in different groups. *N* = 12. Quantified values are mean values ± standard deviation of at least three independent experiments. ^*^ *p* < 0.05, ^**^ *p* < 0.01.

H&E staining was performed to observe the histopathological alterations of lung tissues of rats. The results showed that, in comparison to the neonatal rats treated with air, the number of alveoli in neonatal rats with BPD was decreased and the structure was simplified. Alveolar wall rupture combined with pulmonary bullae, and the ratio of alveolar area/pulmonary septal area increased. After miR-21 antagomir transfection, the number of alveoli in neonatal rats with BPD increased, the lung tissue structure was mature, the alveolar structure was regular, the septal area decreased, and the proportion of alveolar area/pulmonary septal area decreased (Figure 7a–c). Then, to detect vascular development, vWF-positive small blood vessels were tested using immunofluorescence staining. We discovered that the vessel density was reduced in the BPD group and BPD + antagomir NC group (28.32 ± 2.11 vs 12.09 ± 1.03, *p* < 0.01), whereas it was then elevated in the BPD + miR-21 antagomir group (11.54 ± 0.83 vs 20.36 ± 1.46, *p* < 0.05) (Figure 7d and e), indicating that miR-21 depletion could alleviate hyperoxia-induced obstruction of pulmonary vascular development.

## 4 Discussion

The present study reported the hyperoxia-induced increase in miR-21 expression in the pulmonary vasculature *in vitro* and *in vivo* and evaluated the effect of miR-21 on pulmonary vascular responses in BPD. We showed that miR-21 contributed to the hyperoxia-induced degradation of DDAH1, increased ADMA concentration, and decreased eNOS phosphorylation and NO production in human PMVECs, thereby inhibiting PMVEC migration and angiogenesis. Most importantly, we found that miR-21 blockade significantly attenuated the development of hyperoxia-induced BPD in neonatal rats.

Long term exposure to hyperoxia can change the development of the lung and vascular bed, leading to BPD in premature infants [34]. Hyperoxia injury has been confirmed as one of the most common causes of BPD [35]. The pulmonary capillary endothelium is the major target site that is severely damaged by oxygen toxicity [36]. As human infants who die of BPD receive oxygen for more days, their pulmonary vasculature is damaged, and angiogenesis related factors are reduced [37]. Thus, hyperoxia exposure is extensively applied to induce BPD model, leading to the inhibition of distal microvascular formation and the imbalance of pro-angiogenic and anti-angiogenic cytokines in lung development [38]. It is reported that newborn mice treated with hyperoxia display greater arrest in lung alveolarization and angiogenesis [39]. In this study, we established the hyperoxia-induced PMVEC dysfunction *in vitro*, and found that hyperoxia treatment repressed the migratory ability and angiogenesis of PMVECs. Accumulating studies have suggested that miRNAs are promising candidates for novel targeted therapeutic approaches to lung diseases, including BPD [40]. In this study, we found that miR-21 expression in PMVECs was markedly increased by hyperoxia stimulation. MiR-21 is reported to be overexpressed in hyperoxia-mediated BPD [19], which is consistent with our findings. Furthermore, knockdown of miR-21 in hyperoxia-induced PMVECs promoted the migratory ability and angiogenesis ability. Previously, it was reported that miR-21 knockdown could inhibit cell proliferation, migration, and angiogenesis of hypoxic cardiac microvascular endothelial cells [41]. In this study, we believe that knockdown of miR-21 plays a crucial role in promoting angiogenesis in BPD.

During normal lung development, angiogenesis is coordinated accurately, maintaining a balance between cytokines promoting expansion and cytokines promoting the stability of endothelial barrier [42]. Normal alveolar development depends on angiogenesis [43]. Therefore, vascular growth disorder has become a recognized feature of BPD [44]. NO is a signaling molecule involved in modulating vascular tone, vascular remodeling, endothelial permeability, and angiogenesis [45]. eNOS is the main source of vascular NO, expressed in vascular endothelial cells and exerts the crucial function in regulating vascular tone [46]. Its activity is regulated via the phosphorylation of Ser1177 and dephosphorylation of Ser113 [47]. Accumulating studies have confirmed that ADMA is the endogenous NOS inhibitor, and it can repress NOS isoforms, causing diminished bioavailability of NO and endothelial dysfunction [48,49]. In this study, we found that hyperoxia treatment markedly decreased eNOS (ser1177) phosphorylation in PMVECs, while it was then increased by miR-21 knockdown. NO concentration was reduced by hyperoxia treatment and then elevated by miR-21 depletion. Furthermore, hyperoxia treatment elevated the intracellular and extracellular ADMA concentrations in PMVECs. However, miR-21 depletion decreased this effect. Thus, this study confirmed that miR-21 knockdown improved hyperoxia-induced PMVECs dysfunction and promoted angiogenesis via repressing ADMA concentration and elevating NO production. Dihydromyricetin attenuates TNF-α-induced endothelial dysfunction via miR-21-mediated DDAH1/ADMA/NO pathway [50], which further supports our findings.

DDAH has two isoforms, DDAH1 and DDAH2. Most of the ADMA are degraded to citrulline via DDAH or excreted by the kidneys *in vivo* [51]. DDAH1 is the vital enzyme in mediating ADMA in vascular endothelium [52]. Studies have indicated that DDAH depletion is closely correlated with endothelial dysfunction, and it is believed to be the mechanism responsible for ADMA-mediated eNOS impairments [53]. Blueberry anthocyanin‑enriched extract ameliorates transverse aortic constriction‑induced myocardial dysfunction through the DDAH1/ADMA/NO signaling pathway in mice [54]. DDAH1 mediates renal tissue protection in diabetic nephropathy via the ADMA-NOS3-interaction [55]. In this study, we found that DDAH1 was expressed at a low level in hyperoxia-induced PMVECs. Importantly, through prediction of bioinformatics tools and verification of assays, we confirmed that DDAH1 could bind to miR-21 in PMVECs. Furthermore, rescue assays illustrated that DDAH1 knockdown reversed the effects of miR-21 depletion on hyperoxia-induced PMVEC migration, angiogenesis, eNOS phosphorylation, NO production, and ADMA concentration. Previous studies suggested that DDAH1 is vital for regulating NO‐mediated apoptosis and angiogenesis in PMVECs [33]. DDAH1 is conducive to exercise-induced cardiac angiogenesis [56]. DDAH1 may be protective against the development of PH in BPD patients [57]. Furthermore, miR-21- facilitates the pathogenesis of atherosclerosis by regulating DDAH1-ADMA-eNOS-NO pathway [58]. Thus, we concluded that miR-21 knockdown promoted pulmonary angiogenesis in BPD via regulating DDAH/ADMA/NO pathway.

In animal assays, we observed that miR-21 expression was upregulated and DDAH1 expression was downregulated in lung tissues of BPD rats in a time-dependent manner. In addition, miR-21 antagomir restored alveolarization and vascular density in neonatal rats with BPD. Furthermore, Pearson’s correlation analysis indicated that DDAH1 was negatively correlated with miR-21 in lung tissues of BPD rats. It further suggested that miR-21 depletion repressed the progression of BPD.

There are limitations to this study. As miR-21 acts on multiple targets, at this stage we are not yet able to ascribe the benefits of miR-21 inhibition exclusively to upregulation of a specific target or pathway. Although the relative improvements seen in alveolarization and angiogenesis are impressive, more work needs to be performed to define the specific pathways that contribute to these findings. Additionally, although the *in vivo* model we used correlates with the pathological phenotype and long term consequences of neonatal lung disease [59,60], these experiments cannot fully replicate the combination of factors that contribute to the development of BPD. Thus, verifying the efficacy of miR-21 inhibition on BPD treatment is needed in a variety of animal models for future clinical trial. Additionally, our study was carried out to seek answers for acute pathological changes during BPD. Whether miR-21 inhibition has a wide therapeutic time window and exerts long-lasting effects in BPD is the next research direction.

## 5 Conclusion

**Figure 8:**
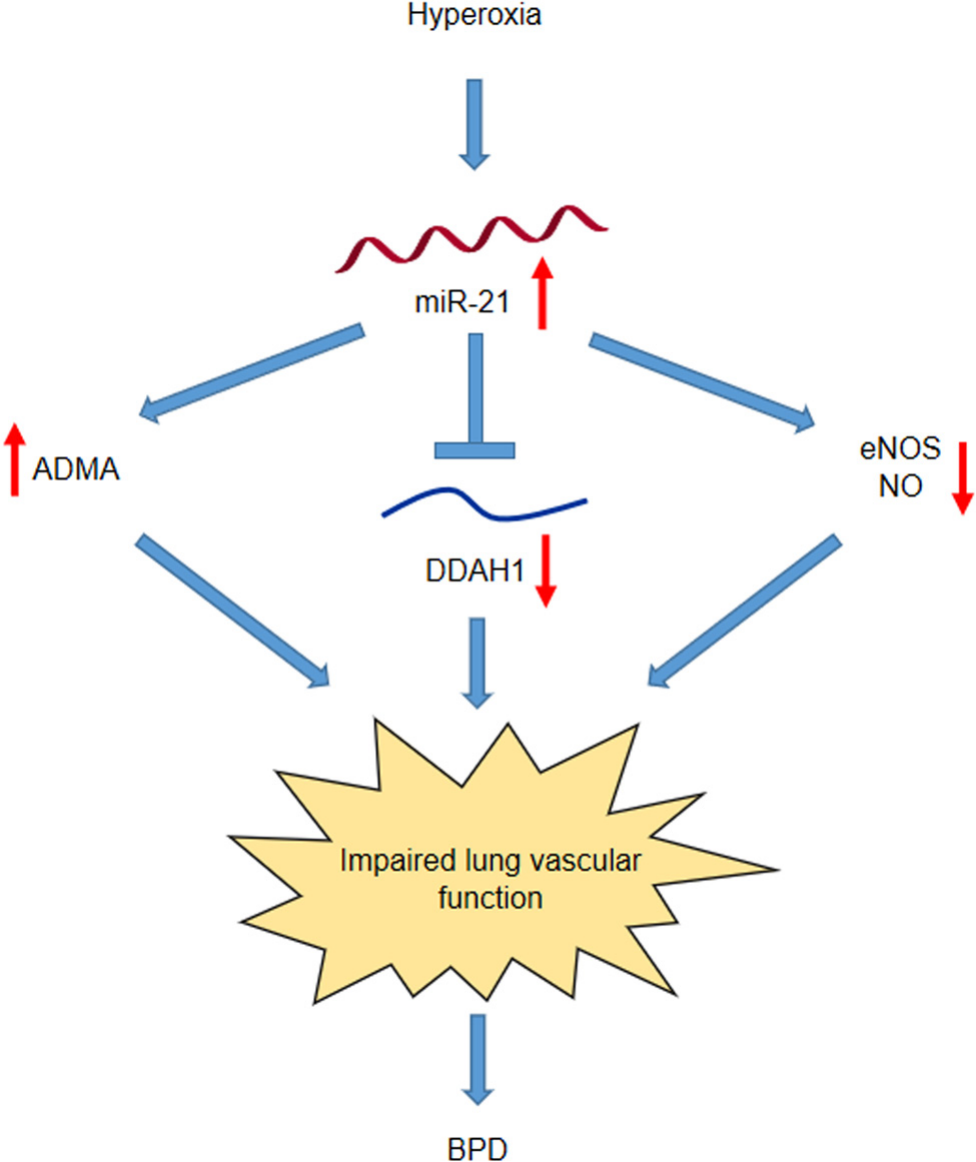
Scientific concept map of miR-21 mediated BPD development through DDAH1/ADMA/NO pathway. Under hyperoxia treatment, miR-21 is upregulated in PMVECs, and miR-21 inhibits DDAH1 expression. It increases ADMA concentration, leading to the decrease in eNOS phosphorylation and NO production. Finally, lung vascular function is impaired and BPD progression is deteriorated.

Taken together, this study proved that inhibition of miR-21 improves pulmonary vascular responses in BPD by targeting the DDAH1/ADMA/NO pathway (Figure 8). Therefore, exploring the contributing mechanisms of miR-21-inhibition might help identify potential therapeutic strategies to improve lung structure and function of patients with BPD.
