# Erratum to “Inhibition of miR-21 improves pulmonary vascular responses in bronchopulmonary dysplasia by targeting the DDAH1/ADMA/NO pathway”

**DOI:** 10.1515/med-2023-0679

**Published:** 2023-03-13

**Authors:** Ying Zhong, Zhiqun Zhang, Xiaoqing Chen

**Affiliations:** Department of Child Health Care, The First Affiliated Hospital of Nanjing Medical University, 368 Jiangdong North Road, Nanjing 210036, Jiangsu, China; Department of Neonatology, Affiliated Hangzhou First People’s Hospital, Zhejiang University School of Medicine, Hangzhou 310000, Zhejiang, China; Department of Pediatrics, The First Affiliated Hospital of Nanjing Medical University, Nanjing 210036, Jiangsu, China

In the published article Zhong Y, Zhang Z, Chen X. Inhibition of miR-21 improves pulmonary vascular responses in bronchopulmonary dysplasia by targeting the DDAH1/ADMA/NO pathway. *Open Med. (Wars)* 2022 Dec 9;17(1):1949–1964. doi: 10.1515/med-2022-0584. PMID: 36561848; PMCID: PMC9743197, authors requested to replace Figures 1, 3, 5, 7 with more representative ones. The changes do not affect the results and conclusions of the study.

**Figure 1:**
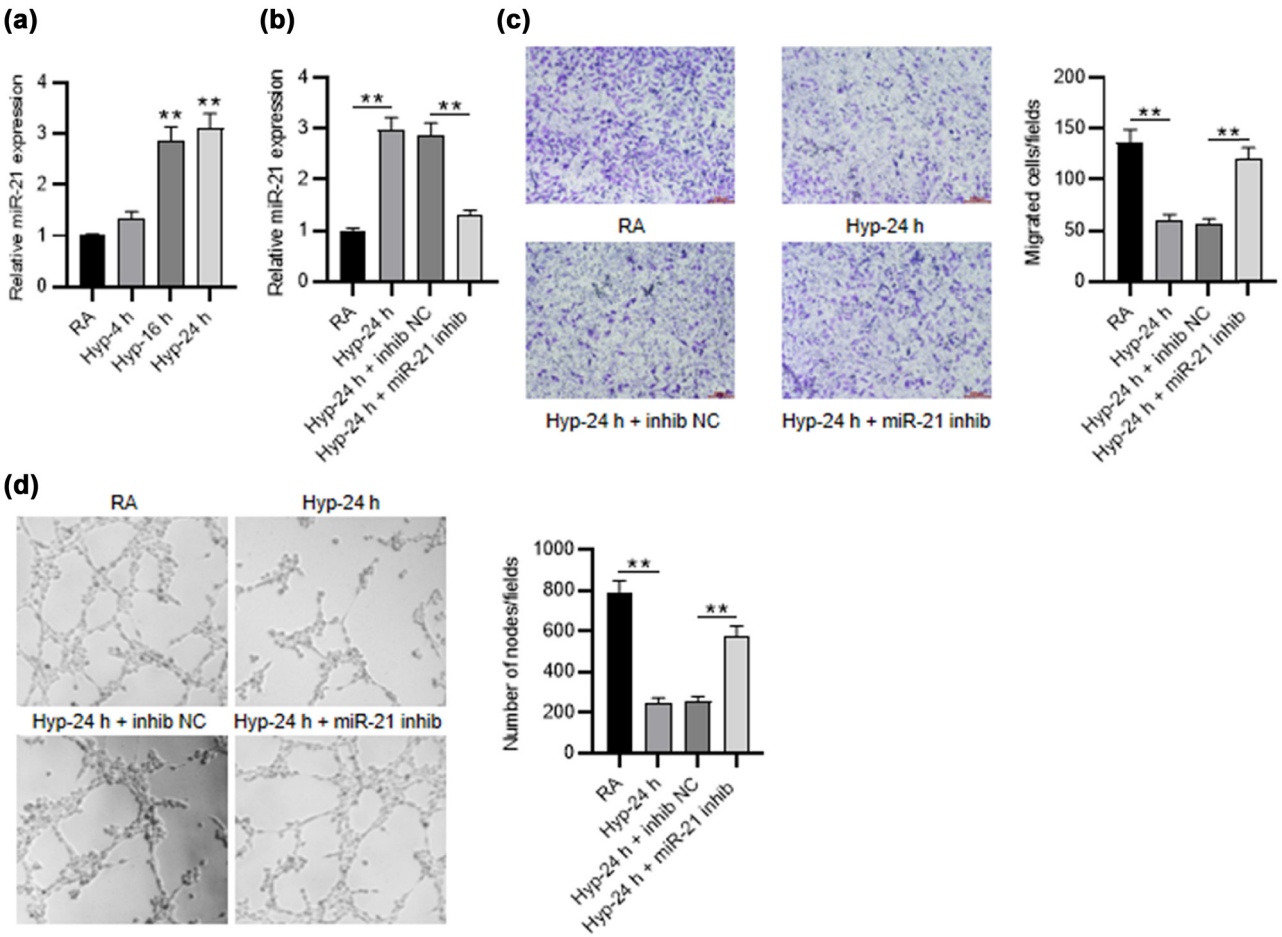
Inhibition of miR-21 improves the angiogenic activity of PMVECs. (a) RT-qPCR was used to measure miR-21 expression in PMVECs treated with RA or hyperoxia (4, 16, and 24 h). (b) The knockdown efficiency of miR-21 inhibitor in PMVECs treated with 24 h of hyperoxia was detected by RT-qPCR. (c) Transwell assay was applied for measuring the migration of hyperoxia-induced PMVECs when miR-21 was silenced. (d) Tube formation assay was performed to estimate the angiogenic activity of hyperoxia-induced PMVECs after miR-21 inhibition. Quantified values are mean values ± standard deviation of at least three independent experiments. **p < 0.01.

**Figure 3:**
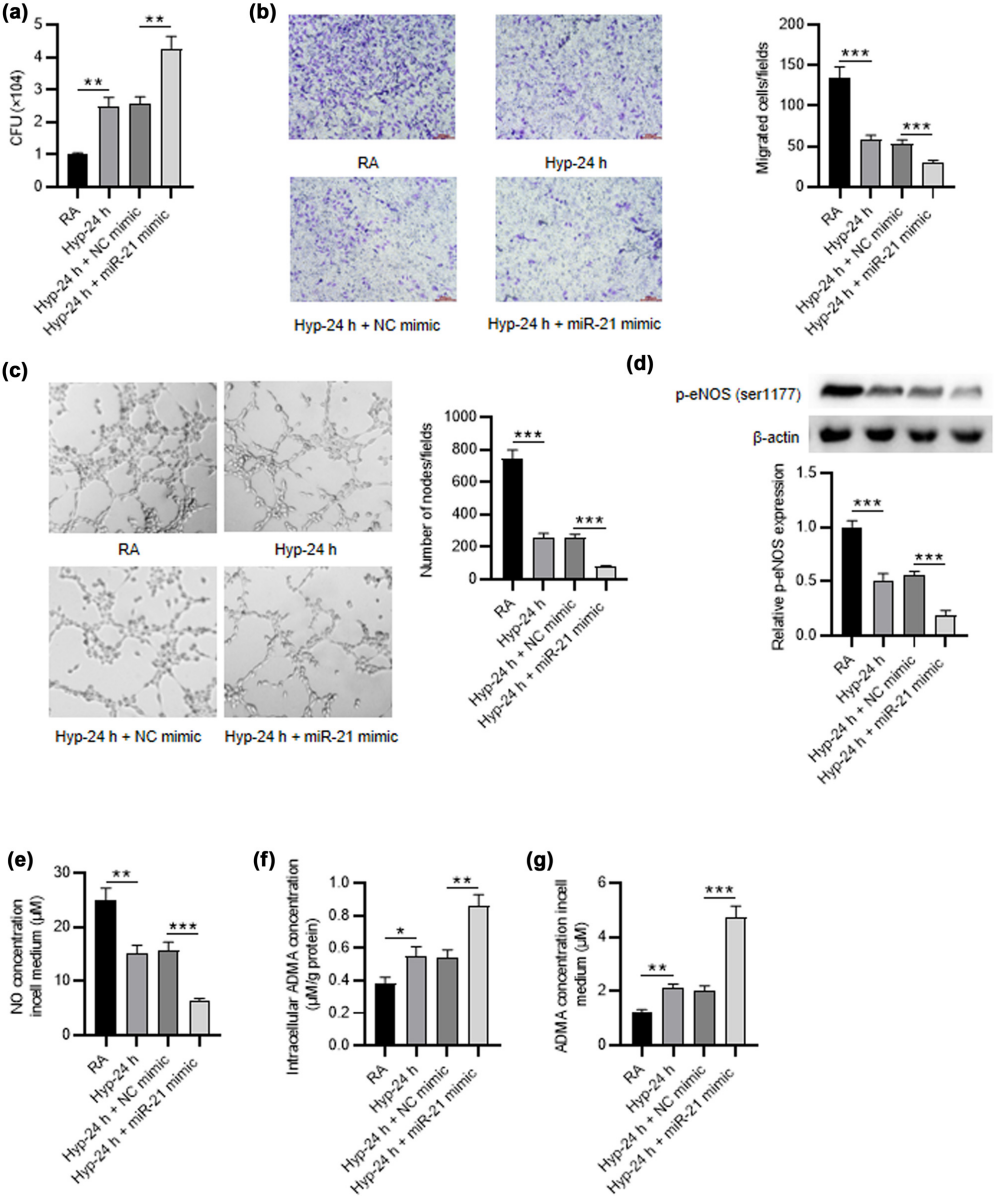
Overexpression of miR-21 strengthens the effects of hyperoxia in PMVECs. (a) The overexpression efficiency of miR-21 mimic in PMVECs treated with 24 h of hyperoxia was detected by RT-qPCR. (b) Transwell assay was applied to detect the migration of hyperoxia-induced PMVECs after miR-21 overexpression. (c) Tube formation assay was performed to estimate the angiogenic activity of hyperoxia-induced PMVECs after miR-21 overexpression. (d) Western blot was utilized to test the p-eNOS (ser1177) level in PMVECs of different groups (RA group, hyperoxia-24 h group, hyperoxia-24 h + NC mimic group, hyperoxia-24 h + miR-21 mimic group). (e–g) ELISA was utilized to detect the NO production and the intracellular and extracellular ADMA concentration in PMVECs of different groups. Quantified values are mean values ± standard deviation of at least three independent experiments. **p < 0.01, ***p < 0.001.

**Figure 5:**
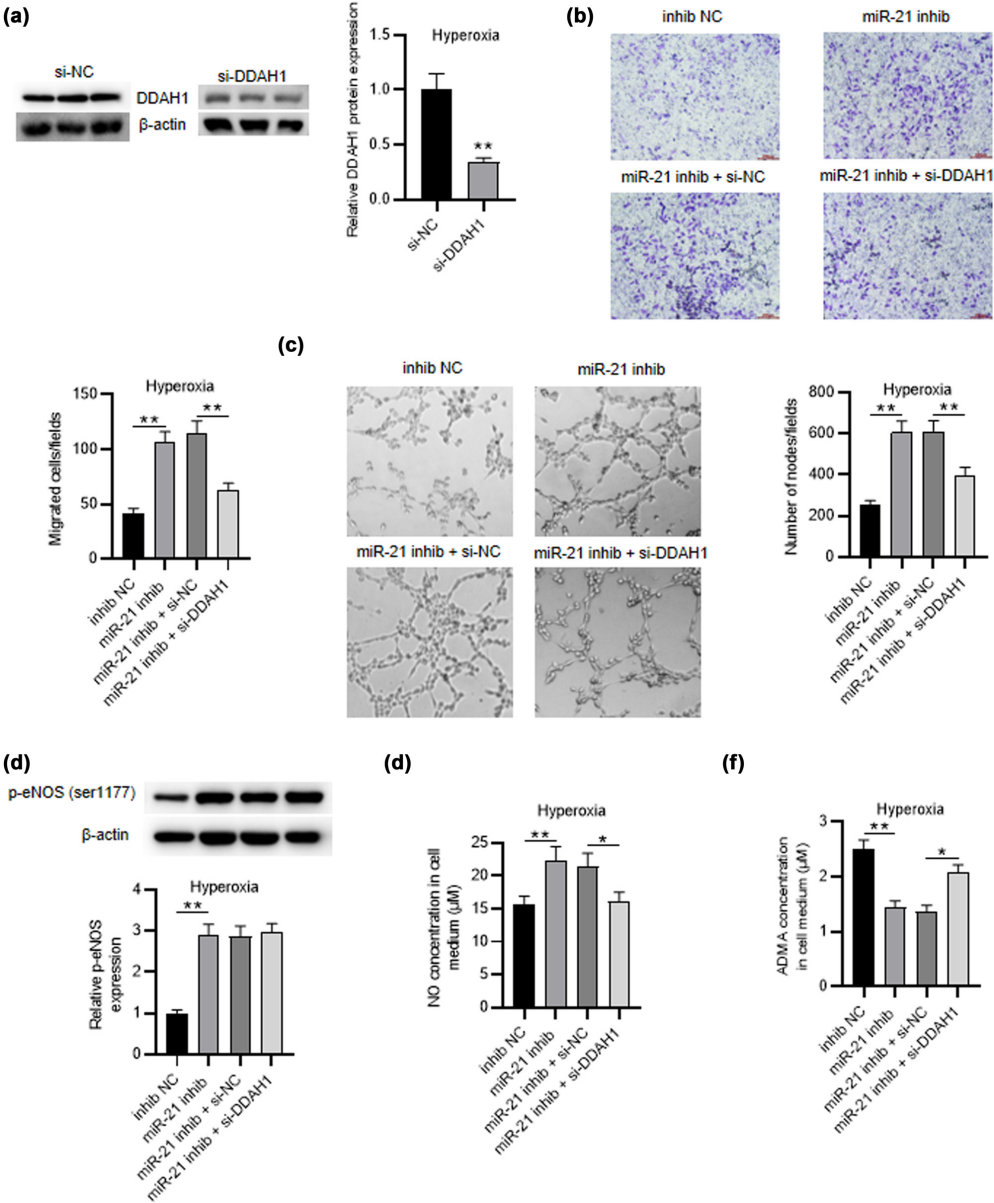
DDAH1 knockdown reverses the effects of miR-21 depletion on hyperoxia-induced PMVEC behaviors, NO production, and ADMA concentration. (a) Western blot was performed to test the knockdown efficiency of si-DDAH1 in hyperoxia-induced PMVECs. (b) Transwell assay was applied for measuring the migration of hyperoxia-induced PMVECs in different groups (NC inhibitor group, miR-21 inhibitor group, miR-21 inhibitor + si-NC group, miR-21 inhibitor + si-DDAH1 group). (c) Tube formation assay was performed to estimate the angiogenic activity of hyperoxia-induced PMVECs in different groups. (d) Western blot was utilized to test the p-eNOS (ser1177) level in hyperoxia-induced PMVECs of different groups. (e and f) ELISA was utilized to detect the NO production and the extracellular ADMA concentration in hyperoxia-induced PMVECs of different groups. Quantified values are mean values ± standard deviation of at least three independent experiments. * *p* < 0.05, **p < 0.01.

**Figure 7:**
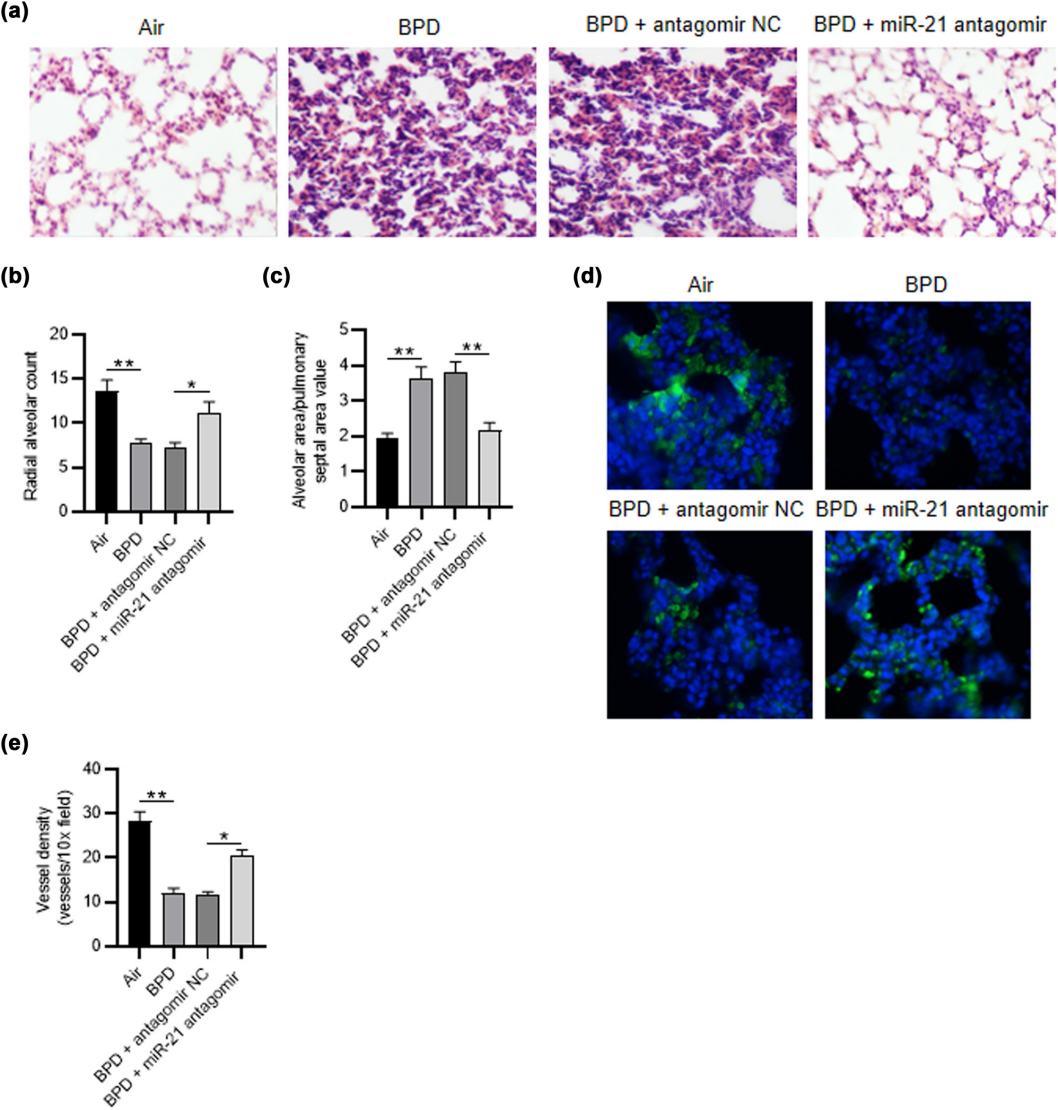
MiR-21 antagomir restores alveolarization and vascular density in neonatal rats with BPD. (a) H&E staining assay was utilized to detect the histopathological alterations of rats in different groups (air group, BPD group, BPD + NC antagomir group, BPD + miR-21 antagomir group). (b and c) The radial alveolar count and the alveolar area/pulmonary septal area value were detected. (d and e) Immunofluorescence staining was applied for measuring the vWF-positive vessels in different groups. *N* = 12. Quantified values are mean values ± standard deviation of at least three independent experiments. * *p* < 0.05, **p < 0.01.

